# Retraction Note: Light Harvesting-like Protein 3 Interacts with Phytoene Synthase and Is Necessary for Carotenoid and Chlorophyll Biosynthesis in Rice

**DOI:** 10.1186/s12284-021-00484-x

**Published:** 2021-05-12

**Authors:** Feng Yang, Das Debatosh, Tao Song, Jian-hua Zhang

**Affiliations:** 1 Co-Innovation Center for Sustainable Forestry in Southern China, College of Biology and the Environment, Nanjing Forestry University, Nanjing, 210037 China; 2 Shenzhen Research Institute, The Chinese University of Hong Kong, Shenzhen, 518057 Guangdong China; 3 Department of Biology, Hong Kong Baptist University and State Key Laboratory of Agrobiotechnology, The Chinese University of Hong Kong, Shatin, Hong Kong, China

**Retraction Note: Rice 14, 32 (2021)**

**https://doi.org/10.1186/s12284-021-00474-z**

The authors have retracted this article. Following publication, errors were discovered in Figure 1, Figure 5 and Table 1. The authors have stated that the phenotypic characterization of oslil3 mutant still needs further experiments to analyze the intermediate metabolites in carotene biosynthesis such as the contents of alfa-carotene and beta-carotene, and that the analysis of oslil3 mutant is not precisely described. In addition, the authors have stated that they need to further analyze the protein accumulation levels of OsPSYs in the mutant as compared with the WT via the PSYs specific antibodies to more accurately validate the interaction between LIL3 and PSYs. The authors intend to resubmit a revised manuscript. All authors agree with this retraction.

